# Biochemical Characterization of Hypothetical Proteins from *Helicobacter pylori*

**DOI:** 10.1371/journal.pone.0066605

**Published:** 2013-06-18

**Authors:** Han-Pil Choi, Silvia Juarez, Sergio Ciordia, Marisol Fernandez, Rafael Bargiela, Juan P. Albar, Varun Mazumdar, Brian P. Anton, Simon Kasif, Manuel Ferrer, Martin Steffen

**Affiliations:** 1 Dept of Biomedical Engineering, Boston University, Boston, Massachusetts, United States of America; 2 Proteomic Facility, CNB-National Centre for Biotechnology, CSIC, Darwin 3, Madrid, Spain; 3 Spanish National Research Council (CSIC), Institute of Catalysis, Madrid, Spain; 4 Bioinformatics Program, Boston University, Boston, Massachusetts, United States of America; 5 New England Biolabs, Ipswich, Massachusetts, United States of America; 6 Dept of Pathology and Laboratory Medicine, Boston University School of Medicine, Boston, Massachusetts, United States of America; Institut Pasteur Paris, France

## Abstract

The functional characterization of Open Reading Frames (ORFs) from sequenced genomes remains a bottleneck in our effort to understand microbial biology. In particular, the functional characterization of proteins with only remote sequence homology to known proteins can be challenging, as there may be few clues to guide initial experiments. Affinity enrichment of proteins from cell lysates, and a global perspective of protein function as provided by COMBREX, affords an approach to this problem. We present here the biochemical analysis of six proteins from *Helicobacter pylori* ATCC 26695, a focus organism in COMBREX. Initial hypotheses were based upon affinity capture of proteins from total cellular lysate using derivatized nano-particles, and subsequent identification by mass spectrometry. Candidate genes encoding these proteins were cloned and expressed in *Escherichia coli*, and the recombinant proteins were purified and characterized biochemically and their biochemical parameters compared with the native ones. These proteins include a guanosine triphosphate (GTP) cyclohydrolase (HP0959), an ATPase (HP1079), an adenosine deaminase (HP0267), a phosphodiesterase (HP1042), an aminopeptidase (HP1037), and new substrates were characterized for a peptidoglycan deacetylase (HP0310). Generally, characterized enzymes were active at acidic to neutral pH (4.0–7.5) with temperature optima ranging from 35 to 55°C, although some exhibited outstanding characteristics.

## Introduction

Our ability to accurately assign gene function lags far behind the tremendous progress made in DNA sequencing. The value of completed sequence would be significantly enhanced if we were able to more completely interpret the complement of genes within genomes. Today, we are typically unable to assign functions to approximately 30% of genes in a new microbial genome, about the same fraction that was not annotated in the first sequenced genome, *Haemophilus influenzae* Rd in 1995 [1]. Given the rapid increases in the production of DNA sequence data associated with microbiomes and meta-genomes, this issue becomes ever more acute.

To address this general issue, we initiated COMBREX (COMputational BRidges to EXperiments, http://combrex.bu.edu) in order to coordinate the efforts of computational and experimental biologists, and to serve as a standard repository for protein function data (predictions, hypotheses and experimental validations) [2]. As part of this effort, COMBREX has: (1) created the Gold Standard Database of experimentally characterized proteins (GSDB) in conjunction with UniProt, NCBI and JCVI [3]; (2) traced the annotated functions of 15% of microbial proteins in COMBREX to their experimental sources; (3) developed a novel gene recommendation system to encourage new experiments for those proteins that would have implications for the largest number of additional proteins; and (4) directly funded the experimental testing of numerous microbial proteins using a novel small-grant model.

Based on a community consensus, COMBREX emphasizes the results from rigorous, direct biochemical experiments, and is therefore willing to entertain hypotheses for specific gene function from the broadest spectrum of sources - experimental, computational, and even “intuition-based” hypotheses based on detailed experience. This mindset was adopted in recognition of the difficulties associated with experimentally approaching the “hypothetical protein.”

*Helicobacter pylori* is a major human pathogen that has been associated with the development of gastritis, gastric ulcers and stomach cancer. Despite its clinical importance, and the large amount of literature devoted to various aspects of its biology, COMBREX documented experimental support for the functions of only 399 of the nearly 1600 proteins-coding genes in *H. pylori*. The annotations of 1082 proteins are based on sequence homology of unknown stringency, and without identification of the original experimental source. Though the number and percentage of experimentally supported gene annotations will increase as the GSDB becomes more comprehensive, it is apparent that *H. pylori* is an appropriate organism for further biochemical characterization.

Several efforts to increase the throughput of biochemical validation have already been quite successful. Yakunin and coworkers [4], [5] developed a set of "entry-level" assays designed to identify the general activity of a protein (phosphatase, dehydrogenase, protease etc.), which is then followed by the use of more specific substrates. A complementary approach uses a mixture of different substrates simultaneously (substrate cocktails) to speed up the characterization of new enzymes [6]. Impressively, Cravatt and coworkers [7], [8] have pioneered the use of “activity-based protein profiling,” enriching enzymes of a particular class using affinity labels and identifying them by mass spectrometry. They and others have applied this technique to 7 classes of enzymes.

In this work, we utilized new methodology (to be described in detail in a forthcoming manuscript) to generate hypotheses. The method utilizes nano-particles coated with substrate analogs to enrich proteins from cell lysates of *H. pylori*. The focus on this paper is the biochemical characterization of the enriched proteins. The isolated proteins were examined in two ways. First, an aliquot of the isolated material was digested with trypsin, and analyzed by mass spectrometry. A second aliquot of isolated protein was utilized in biochemical assays to confirm suspected activities. The combined results at this stage provide putative functions and probable protein identities, and were treated as hypotheses, which we then explored using standard recombinant DNA technology and traditional *in vitro* biochemistry: the proteins identified by mass spectrometry were cloned, expressed in *Escherichia coli*, purified using an epitope tag and subjected to in vitro biochemical analysis.

## Materials and Methods

### Chemicals and strains

Chemicals, biochemicals and solvents used for enzymatic tests were of the purest grade available and were purchased from Sigma-Aldrich Co. (St. Louis, MO). *H. pylori* ATCC 26695 was grown on Trypticase Soy Agar (TSA) plates at 37°C, under 5% CO_2_ for 48 hours. Protein lysates were generously provided by Dr. Xuesong Zhang and Dr. Martin Blaser using the BugBuster Protein extraction reagent (Novagen; Darmstadt, Germany), and stored at a protein concentration above 0.4 mg ml^−1^ at –80°C until use.

### Protein enrichment via affinity purifications

As the focus of this paper is the *in vitro* biochemical characterization of recombinant proteins, a full description of these methods will be described in detail in a forthcoming publication. Briefly, total cell lysates containing 10 µg total protein in 100 µl phosphate buffered saline (PBS) were incubated for 5 mins with 10 µg gold nano-particles coated with various substrate analogs. Nano-particles were produced as indicated [9]. Nano-particles (∼10 µg in 100 µl of PBS) were mixed with 100 µl of a solution containing potential substrates (0.2 mg ml^−1^ in PBS) in closed vials and incubated overnight at 4°C in an orbital shaker. The substrates utilized were adenine, acetylated xylan [10], guanosine-5'-triphosphate (GTP)/dihydroneopterin triphosphate (both at equal amount), succinyl-Ala-Ala-Ala-*p*-nitroanilide, cyclic adenosine monophosphate (cAMP) and GTP. The suspension was then concentrated using centrifugal evaporation under vacuum. Coated nano-particles were stored at –20°C until use.

Protein lysate and coated nano-particles were incubated for 5 min, allowing proteins to bind the potential substrates. Nano-particles with captured protein(s) were separated by ultrafiltration through low-adsorption hydrophilic 500000 nominal molecular weight limit (NMWL) cutoff membranes (regenerated cellulose, Amicon), followed by a wash step with PBS (1∶20 diluted). Proteins which bind the attached substrates with high affinity are expected to be isolated. We have observed that those proteins that are most efficiently captured or those with high affinity (K_m_ below 2 mM) and modest reaction rates (lower than 50 min^−1^), while proteins with higher reaction rates are captured somewhat less efficiently. The nano-particles with their bound protein were dried under vacuum, and stored at –20°C. They were then analyzed in two ways. (1) For protein identification, nano-particles were incubated with trypsin (Roche, sequencing grade) overnight at 37°C. Tryptic peptides were collected by ultrafiltration through 10 kDa cutoff membranes, and evaporated to dryness. Peptides were analyzed using RP-LC-MALDI TOF/TOF mass spectrometry (see details below). (2) For biochemical testing of isolated protein material, proteins are recovered from the nano-particles after incubation in PBS for 24 h at 4°C. No degradation in enzymatic activity was observed, when compared to shorter incubations for test enzymes. Released proteins were separated from nano-particles by ultra-filtration through low-adsorption hydrophilic 500000 NMWL cutoff membranes. Proteins were purified by high-performance gel filtration chromatography (FPLC) using a 25 ml Superose-12 pre-packed HR 10/30 gel-filtration column and PBS buffer (0.4 ml min^−1^). Protein solutions, at concentrations of at least 0.1 µg ml^−1^, were stored at –20°C. A full description of the affinity capture methodology will be presented elsewhere. The high specificity of the protein capture method can be observed in Figure S1.

### MALDI peptide mass fingerprinting, MS/MS analysis and database searching

Proteins were analyzed essentially as in [11]. Briefly, trypsin, proteomics grade (Sigma) in 25% acetonitrile (ACN)/50 mM ammonium bicarbonate solution was added to the protein solution, and reactions were stopped after 4 hours by adding 0.5% trifluoroacetic acid (TFA). Tryptic eluted peptides were dried by speed-vacuum centrifugation and resuspended in 4 µl of MALDI solution (30% ACN/15% isopropanol/0.1% TFA). A 0.8 µl aliquot of each peptide mixture was deposited onto a 386-well OptiTOF^TM^ Plate (AB SCIEX, Foster City, CA, USA) and allowed to dry at room temperature. A 0.8 µl aliquot of matrix solution (3 mg/mL α-cyano-4-hydro-cinnamic acid in MALDI solution) was then deposited onto dried digest and allowed to dry at room temperature. For MALDI-TOF/TOF analysis, data was automatically acquired using an ABI 4800 MALDI TOF/TOF mass spectrometer (AB SCIEX, Foster City, CA, USA) in positive ion reflector mode. Spectra were smoothed and corrected to zero baseline using ABI 4000 Series Explorer Software v3.6. Spectra were internally calibrated with the mass signals of trypsin autolysis to achieve a typical mass measurement accuracy of <25 ppm. Spectra were analyzed using MASCOT software v.2.2.04 (Matrix Science, London, UK) using a custom protein database containing all possible *H. pylori* protein sequences. The mass tolerance for precursors was set to ± 50 ppm, and to ± 0.3 Da for MS/MS fragment ions. Peptide identifications were accepted when scored at greater than 95.0% probability by the Mascot algorithm [12]. Protein identifications were treated as hypotheses for further biochemical characterization.

### Cloning, expression and purification of recombinant proteins

Genomic DNA was extracted from *H. pylori* ATCC 26695 by DNA Maxi Kit (Qiagen) according to manufacturer’s protocol. The recombinant plasmids for the (His)_6_-tagged proteins were constructed by PCR amplification of the corresponding gene from *H. pylori* ATCC 26695 genomic DNA. The oligonucleotide primers used for PCR were designed to complement the TOPO-Cloning system from Invitrogen or In-Fusion Cloning system from Clontech and were as follows: HP0267 (Fwd 5′-CAC CAT GCA AGA AAT CAT AGG AGC GTC-3′ and Rev 5′-TTA GAT CAC CCT TTT CCC CCC TAA AAA C-3′), HP0310 (Fwd 5′-CAC CAT GGC AAA AGA AAT TTT AGT GG-3′ and Rev 5′-CTA TTT TTT TCT AGG GTT TCG-3′), HP0959 (Fwd 5′-ACC ACG GTG GTC ATA TGG CGT TAG TTA AGG AAG TGT TGG TAG-3′ and Rev 5′-GTT AAC CTT ACT CGA GTT AAA TGA TTT GCA AGG GGT TTT TAA AAT TCT C-3′), HP1037 (Fwd 5′-CAC CAT GAA AGG ATT AGA AAG AGA ATC G-3′ and Rev 5′-TCA CAA AAG CTC AGA CCT AGA-3′), HP1042 (Fwd 5′-CAC CAT GAT GCA AGT TTA CCA CCT TTC-3′ and Rev 5′-TTA AGC GTT GTT GAA GAT TTC-3′), and HP1079 (Fwd 5′-CAC CAT GAT TCA GTC TGT TCG CAT C-3′ and Rev 5′-TTA ACC AAA AAG ATT CTC TTC-3′). The amplified gene inserts were cloned into the pET200/D-TOPO vector using the Champion pET Directional TOPO Expression Kit from Invitrogen, or into the pCOATexp mH6 vector (a gift from Shaorong Chong, New England Biolabs) after digestion with *Nde*I and *Xho*I using the In-Fusion HD cloning. Recombinant plasmids were transformed into One Shot TOP10 Chemically Competent *E. coli* (Invitrogen) or Stellar Competent *E. coli* (Clontech). From the transformed *E. coli*, recombinant plasmids were purified by QuickClean II Plasmid Miniprep Kit (GenScript), and sequenced by GENEWIZ to confirm the identity of the construct. The purified recombinant plasmids for the (His)_6_-tagged proteins were transformed into BL21(DE3) Competent *E. coli* (New England Biolabs) or T7 Express *lysY/I^q^* Competent *E. coli* (New England Biolabs). Single colonies were grown overnight in LB broth containing ampicillin or kanamycin at 37°C. Larger volume cultures were inoculated from the overnight growth. When these cultures achieved an OD_600_ value of ∼0.6, recombinant protein expression was induced by 0.5 mM isopropyl-β-D-thiogalactopyranoside (IPTG). After additional incubation at ∼20°C, cells were harvested and stored at –80°C. The frozen cell pellets were resuspended in lysis buffer (50 mM Tris-HCl, pH 8.0, 300 mM NaCl, 10 mM imidazole, 10% glycerol, 5 mM β-mercaptoethanol) containing EDTA-free Protease Inhibitor Cocktail (Roche), lysed via sonication, and then 0.1% Triton X-100 was added. Recombinant proteins were purified from the cell-free supernatant by using Ni-NTA agarose (Qiagen). After washing, recombinant proteins were eluted with imidazole (50 mM Tris-HCl, pH 8.0, 300 mM NaCl, 250 mM imidazole, 10% glycerol, 5 mM β-mercaptoethanol, 0.1% Triton X-100). The purified proteins were analyzed by SDS-polyacrylamide gel electrophoresis. Protein concentration was determined using the Bio-Rad Bradford Protein Assay.

### Biochemical characterization

Biochemical characterization was performed twice, once on isolated protein material and once on recombinant purified protein. *K*_m_, V_max_, and *k*_cat_ values were determined using nonlinear regression to fit the values for initial velocity and substrate concentration to the Michaelis-Menten equation. Unless otherwise indicated, the pH dependence of a reaction was tested in the range of pH 4.0–9.5, and the temperature dependence in the range of 30–70°C. The buffers used were: citrate (pH 4.0–5.0), acetate (pH 5.0–6.0), 2-(*N*-morpholino) ethanesulfonic acid (MES) (pH 6.0–7.0), 4-(2-hydroxyethyl)piperazine-1-ethanesulfonic acid (HEPES) (pH 7.0–8.0), Tris-HCl (pH 8.0–9.0) and glycine (pH 9.0–9.5), all at 100 mM. The pH was adjusted at 25°C. In all cases, one unit of the enzyme was defined as the amount of enzyme that catalyzed the formation of 1 µmol of reaction product per minute at 30°C. Three independent experiments were performed for each parameter and graphs were plotted using mean values and standard deviations wherever appropriate. In all cases, for the characterization of isolated protein material, the absorbance was measured using a BioTek Synergy HT spectrophotometer. The following assays were performed.

*1. Acetyl esterase* (HP0310) – Esterase kinetic parameters were initially assayed using *p*-nitrophenyl (*p*NP) esters [13] with minor modifications. Briefly, kinetic parameters were calculated by adding 0–12 nM enzyme solution and 0–2 mM *p*NP ester to 190 µl buffer. The reaction was followed spectrophotometrically measuring the release of *p*-nitrophenol at 405 nm. Substrates tested included *p*NPacetate, *p*NPpropionate and *p*NPbutyrate. The standard acetyl esterase assay was performed in 100 mM sodium acetate buffer, pH 6.0 and 30°C. Under our experimental conditions, the absorption coefficient for *p*NP was measured as 3021 M^−1^ cm^−1^. For other pHs, the absorption coefficient was calculated. The release of acetate from acetylated polymeric substrates chitin, chitosan and xylan was measured using phenol red as pH indicator [14]. Each well in a 96-well microtiter plate contained 0–12 nM enzyme solution, 0–100 mg ml^−1^ substrate, and 0.911 mM phenol red in 200 µl of 5 mM *N*-(2-hydroxyethyl)piperazine-*N*’-(3-propanesulfonic acid (EPPS) buffer (pH 8.0). The acetate release was colorimetrically monitored at 550 nm. Stock solutions (500 mg ml^−1^ in dimethylsulfoxide, DMSO) of substrates were prepared immediately prior to use. Under our experimental conditions, the absorption coefficient for phenol red was measured as 8450 M^−1^ cm^−1^. Acetylated xylan and chitosan were prepared by incubating the substrates with pyridine:acetic anhydride (1:1) at 25°C for 48 hrs followed by the recovery of acetylated products by evaporation with ethanol [10]. The standard acetyl esterase assay contained [E]_o_ =  12 nM, [acetylated xylan]  =  10 mg ml^−1^, 5 mM EPPS buffer, pH 8.0, 0.45 mM phenol red in a total volume of 200 µl, at 30°C.

*2. Adenosine deaminase* (HP0267) – The deamination of adenine or adenosine was determined in a 96-well microtiter plate using a coupled assay with glutamate dehydrogenase (GDH; Roche Applied Science, Mannheim, Germany) [15], in which the formation of ammonia was followed at 340nm in the presence of 0–12 nM enzyme solution, 0–2 mM adenine or adenosine, 0.15 mM NADH, 25 mM R-ketoglutarate and 5 µg ml^−1^ GDH in a final volume of 200 µl. The standard adenosine aminohydrolase assay contained [E]_o_ =  12 nM, [adenosine] 1 mM, [NADH] 0.15 mM, [R-ketoglutarate] 25 mM, [GDH] 5 µg ml^−1^, 100 mM HEPES (pH 7.5), at 30°C. For purified recombinant protein, biochemical activity was assayed using two methods, the first being the coupled GDH assay, and second by following the decrease of absorbance of the substrate adenosine at 265 nm using Infinite M 200 PRO (TECAN) in the presence of 50 mM potassium phosphate, pH 7.6, and 0.045 mM adenosine or adenine. Kinetic parameters were determined at 25°C using 90 nM of enzyme and various concentrations of adenosine (ranging from 0 to 1 mM) [16], [17].

*3. Aminopeptidas*e (HP1037) – Enzyme activities toward amino acidic derivatives (Xaa) of *p*-nitroanilide (Xaa-*p*NA) derivatives were determined in a 96-well microtiter plate by adding an enzyme solution (0–20 nM) and a substrate solution in DMSO (1–20 mM) in a final volume of 200 µl and continuously monitored the increase in absorption at 405 nm caused by the release of *p*-nitroaniline [18]. The initial activity rate was determined from the linear part of the optical density profile (ε_405nm_ of 10600 M^−1^ cm^−1^). The hydrolysis of Phe-Arg-methylcoumarine amide and succinyl-Leu-Leu-Val-Tyr-methylcoumarine amide was recorded under the same conditions, but monitoring the free amino methyl coumarine fluorimetrically (λ_ex_ = 380 nm and λ_em_ = 460 nm). Buffer was supplemented with 5 mM calcium chloride and 0.1% w v^−1^ Brij. The standard aminopeptidase assay contained [E]_o_ =  12 nM, [N-succinyl-Ala-Ala-Ala-Pro-Phe-*p*-nitroanilide] 3 mM, 100 mM sodium citrate (pH 4.5), at 30°C. For purified recombinant protein, the substrate tested was N-Succinyl-Ala-Ala-Pro-Phe-*p*-nitroanilide and the buffer used was 100 mM citrate buffer, pH 4.5, the observed optimum for the isolated material. All other aspects of the assay were identical.

*4. ATPase/GTPase activity* (HP1079) − The hydrolysis of nucleotide triphosphates (NTP) was tested in 200 µl mixtures containing enzyme solution (0–20 nM), NTP (0–5.0mM), 10 mM MgCl_2_, and 10% glycerol (v v^−1^) [19]. Reactions were incubated at the indicated temperature and pH for 30 min, after which reactions were stopped by the addition of 80 µl of a color reagent (3∶1 ratio of 0.045% malachite green and 4.2% ammonium molybdate in 4 N HCl) and 100 µl of 34% citric acid solution. After incubation at room temperature for 30 min, the absorbance was measured at 660 nm. The standard NTPase assay contained [E]_o_ =  12 nM, [ATP] 3 mM, 100 mM sodium acetate (pH 6.0), at 30°C. For purified recombinant protein, optimal ATP hydrolysis was observed 20 mM Tris-HCl (pH 8.0), 0.57 mM EDTA, 5 mM MgCl2, 133 mM NaCl, 3 mM KCl, 3 mM ATP. The mixture containing enzyme was incubated at 37°C for 1 hr. Liberated P_i_ was assayed with Malachite Green Phosphate Assay Kit (BioAssay Systems) according to manufacturer’s protocol [20], [21].

*5. GTP cyclohydrolase* (HP0959) – The conversion of GTP to dihydroneopterin triphosphate, cyclohydrolase enzymatic activity, was tested in a 96-well microtiter plate with 500 µM GTP, 2.5 mM ethylenediaminetetraacetic acid (EDTA), 0.05 M KCl, and 10% glycerin (v v^−1^) in 100 µl. Reactions were carried out for 2 hrs in darkness. The reaction was terminated by adding 1.1 ml of the above buffer (lacking GTP) and 100 µl ml of an acidic iodine solution (1% I_2_ and 2% potassium iodide in 1 N HCl) [22]. After 15 min incubation at room temperature, the mixture was centrifuged and the excess of iodine was eliminated from the supernatant by the addition of 100 µl of 2.0% ascorbic acid (in the above buffer), followed by the addition of 120 µl of 1 N NaOH and alkaline phosphatase (12.5 units; Roche Applied Science, Mannheim, Germany) at 37°C for 100 min. Reaction product was detected spectrofluorometrically: λ_ex_ of 350 nm and λ_em_ of 450 nm [22]. The standard GTP cyclohydrolase assay contained [E]_o_ =  12 nM, [GTP] 0.2 mM, 100 mM sodium citrate (pH 4.0), at 30°C. For purified recombinant protein, cyclohydrolase activity was measured by monitoring absorbance at 330 nm, with the following reaction conditions: 0.2 mM GTP in 100 mM Tris-HCl, pH 8.0, 100 mM KCl, and 2.5 mM EDTA [23].

*6. Phosphodiesterase* (HP1042 assay) *–* Phosphodiesterase activity was measured in a 96-well microtiter plate by following the hydrolysis of the chromogenic substrate bis-*p*-nitrophenyl phosphate (bis-*p*NPP) in a reaction mixture containing enzyme solution (0-20 nM) and a substrate solution (1–5 mM), and 1.0 mM MnCl_2_ in a final volume of 200 µl, at 410 nm. The measurement of phosphodiesterase activity against several 2′,3′- and 3′,5′-cyclic nucleoside monophosphates was based on the alkaline phosphatase-sensitive nucleotide product as follows: 200 µl mixtures containing 0–5 mM of substrate, and 0.1 mM MnCl_2_. After 20 min incubation at the indicated temperature and pH with enzyme solution (0–20 nM), 1 unit of alkaline phosphatase (Roche Applied Science, Mannheim, Germany) was added and the reaction was incubated for an additional 10 min at 30°C. Liberated P_i_ was assayed with malachite green reagent, as described above. The standard phosphodiesterase assay contained [E]_o_ =  12 nM, [bis-*p*NPP] 2.0 mM, 100 mM sodium citrate (pH 4.5), at 30°C.

## Results

Total protein lysate was prepared from *H. pylori* ATCC 26695 grown on TSA plates, and the clarified lysate was incubated with six substrate-coated nano-particles as described in Materials and Methods. Captured proteins were digested with trypsin and analyzed by mass spectrometry, or, alternatively isolated from nano-particles and tested for biochemical activities suggested by the attached substrates. For those cases in which appropriate biochemical activity was observed, the genes encoding the proteins identified by mass spectrometry were cloned into a vector containing sequence for an N-terminal His-tag epitope using standard recombinant DNA methodology. Proteins were expressed in *E. coli* BL21(DE3), and purified using immobilized metal affinity chromatography. Purified recombinant protein was tested for the biochemical activity initially observed. The detailed functional roles of six previously unannotated proteins are described below.

**Figure 1:**
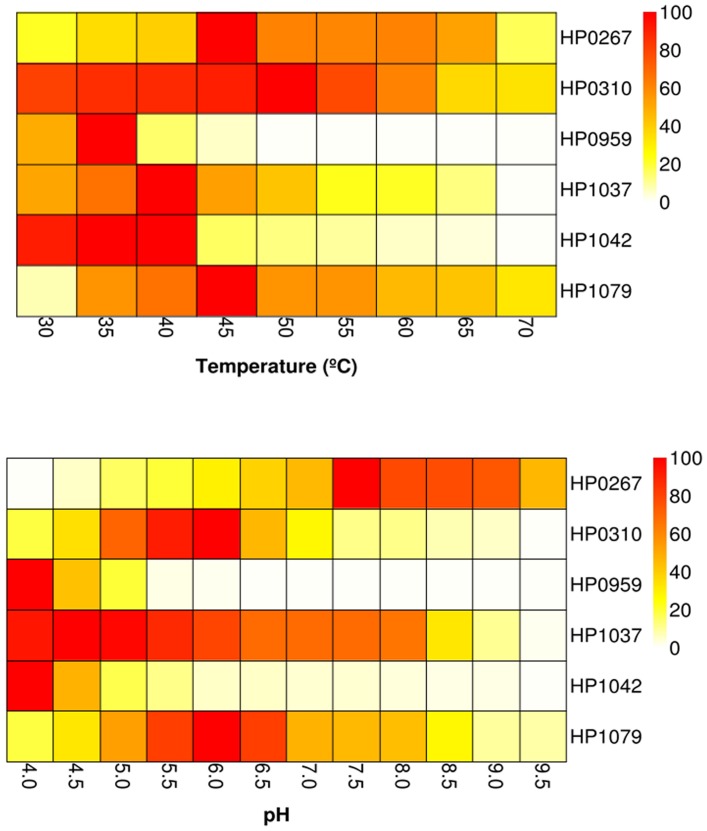
Optimal temperature (upper) and pH (lower) for hypothetical proteins from *H.* *pylori*. The heat map colors represent the relative percentages of activity (in terms of *k_cat_*) as compared to the maximum (100%) within each enzyme. k_cat_ values were determined using nonlinear regression to fit the values for initial velocity and substrate concentration to the Michaelis-Menten equation as described in Materials and Methods. The pH dependence of a reaction was tested in the range of pH 4.0–9.5 at 30°C, and the temperature dependence in the range of 30–70°C at the optimal pH (4.0 for HP0142 and HP0959; 6.0 for HP0179 and HP0310; 7.5 for HP0267; and 4.5 for HP1037). The buffers used were: citrate (pH 4.0–5.0), acetate (pH 5.0–6.0), MES (pH 6.0–7.0), HEPES (pH 7.0–8.0), Tris-HCl (pH 8.0–9.0) and glycine (pH 9.0–9.5), all at 100 mM. Reaction conditions: [E]_o_ =  0–12 nM, [substrate: HP0310 (*p*NPacetate and acetylated xylan), HP0267 (adenosine), HP1037 (N-succinyl-Ala-Ala-Ala-Pro-Phe-*p*-nitroanilide), HP1079 (ATP), HP0959 (GTP), HP1042 (bis-*p*NPP)] ranging from 0 to 20 mM. Three independent experiments were performed for each parameter and graphs were plotted using mean values.

*HP0959* - HP0959 is annotated as a “hypothetical protein” with a predicted mass of 26.8 kDa. This protein was isolated using nano-particles coated with both GTP and dihydroneopterin triphosphate, and it was subsequently tested for its ability to hydrolyze GTP to form dihydroneopterin triphosphate. The protein did show a high affinity (*K*_m_ of 0.040±0.005 mM) and activity (*k_cat_* of 2593±29.0 min^−1^) for the substrate (Table 1); no product was detected when GTP was replaced by GDP, GMP, ATP, CTP and UTP. HP0959 was observed to have peak activity at about 35°C, and approximately at pH 4.0, with a strong decrease above those values (Figure 1). Biochemical activity was also confirmed using the method of [23] by monitoring absorbance at 330 nm. Therefore, HP0959 should be considered as a “GTP cyclohydrolase l” (EC 3.5.4.16).

**Table 1:** Kinetic parameters of native pure proteins directly isolated from *Helicobacter pylori*.

Enzyme	Substrate^a^	K_m_ (mM)^bc^	V_max_ (μmol ml^−1^ min^−1^)	k_cat_ (min^−1^)	k_cat_/K_m_ (s^−1^ mM^−1^)
**HP0267**					
	Adenosine (Native protein)	0.32±0.19 (0.032–2)	33.75±3.99	1101±151 (0.3–10800)	57.3 (30–150)
	Adenine (Native protein)	2.04±0.16 (0.0147–6.6)	0.003±0.001	0.47±0.13 (330–804)	0.004
	6-Chloroadenine	n.d.	n.d.	n.d.	
	Adenosine (Recombinant protein)	1.29±0.24 (0.0147–6.6)	0.03±0.001	331±10 (0.3–10800)	4.28 (30–150)
**HP0310**					
	Chitin	0.85±0.14	6.90±0.78	775±58	15.2
	Chitosan	0.85±0.11	5.01±0.21	968±13	18.9
	Acetylated xylan	0.41±0.03	73.7±4.5	2549±89	103
	*p*NPacetate	0.18±0.05 (0.00186–0.9)	37.4±1.7	1209±55 (2856)	112
**HP0959**					
	GTP	0.040±0.005 (0.004–980)	1.42±0.06	2593±29 (0.066–0.21)	1080
**HP1037**					
	NSAAAPPpNA	1.38±0.09	18.2±1.5	1145±39	13.8
	PGPApNA	7.56±0.27	1.42±0.06	23.2±0.6	0.05
	SAAApNA	1.12±0.30	1.54±0.13	122±5	1.8
	PAMCA	7.43±0.36	0.75±0.09	10.2±0.4	0.02
	SLLVTMCA	4.06±0.13	0.78±0.07	4.6±0.2	0.02
**HP1042**					
	bis-*p*NPP	0.53±0.04	1.13±0.10	1085±12	34.1
	cAMP	0.88±0.12 (0.000079–7)	2.01±0.07	2008±73 (0.0558–34020)	38
	cGMP	0.82±0.15 (0.00002–1)	5.13±0.20	957±82 (252–40020)	19.5
**HP1079**					
	ATP	1.02±0.05 (0.001–0.98)	319±21	9.3±1.2 (1.14–7980)	0.15
	GTP	0.58±0.05 (0.0008–0.85)	122±11	0.57±0.12 (0.108–1260)	0.02

a Abbreviations: bis-*p*NPP, bis-*p*-nitrophenyl phosphate; NSAAAPPpNA, N-succinyl-Ala-Ala-Ala-Pro-Phe-*p*-nitroanilide; PGPApNA, pyroglutamyl-Pro-Arg-p-nitroanilide; SAAApNA, succinyl-Ala-Ala-Ala-*p*-nitroanilide; PAMCA, Phe-Arg-methylcoumarine amide; SLLVTMCA, succinyl-Leu-Leu-Val-Tyr-methylcoumarine amide.

b Kinetic parameters determined at 30°C as described in Figure 1 legend and Materials and Methods.

c Previously reported numbers for the kinetic parameters are obtained from Brenda [26], and listed in parentheses.

*HP1037* - HP1037 is annotated as a “hypothetical protein” with a predicted mass of 40.8 kDa, and with two identified domains, Creatinase_N and Peptidase_M24 (Pfam). HP 1037 has previously been predicted as an aminopeptidase based on sequence homology to characterized proteins [24]. This protein was isolated using nano-particles coated with succinyl-Ala-Ala-Ala-*p*-nitroanilide. As shown in Table 1, the protein was able to cleave five different aminopeptides tested, with N-succinyl-Ala-Ala-Ala-Pro-Phe-*p*-nitroanilide being preferred. This result can be explained by the up to 9-fold greater *k*_cat_ value and the up to 5.5-fold lower *K*_m_ value for this substrate. Both, *p*-nitroanilide (PNA) and methylcoumarine amide derivatives were accepted as substrates, with substrates containing multiple alanines (*i.e.* N-succinyl-Ala-Ala-Ala-Pro-Phe-*p*-nitroanilide and succinyl-Ala-Ala-Ala-*p*-nitroanilide) being preferred (Table 1): *k*_cat_ values from one to two orders of magnitude higher. In addition, further tests of enzyme activity with different peptide substrates suggest cleavage specificity for peptides containing the following amino acids, Ala, Pro, Phe, Arg Phe, Leu, Val and/or Tyr. With N-Succinyl-Ala-Ala-Ala-Pro-Phe-p-nitroanilide, HP1037 demonstrated maximum activity at pH 4.0–5.0 and about 40°C. Therefore, HP1037 should be considered an “aminopeptidase” (EC 3.4.11.-).

*HP1042* - HP1042 is annotated as a “hypothetical protein” with a predicted mass of 40.2 kDa, and one domain DHH phosphatase family (Pfam). The protein was isolated using nano-particles coated with cAMP. The protein showed significant activity (*k_cat_* of 1085±11.7 min^−1^) towards the synthetic substrate bis-*p*-nitrophenyl phosphate (bis-*p*NPP), a general substrate for phosphodiesterases and nucleases. The biochemical activity of HP1042 against naturally occurring phosphodiesters such as cyclic nucleotides and phospholipids was tested further. No phosphohydrolase activity was found toward various phosphatidylcholine and phosphorylated sugars, but it was observed for the cyclic nucleotides cAMP and cyclic guanosine monophosphate (cGMP) (Table 1), with a (*k*_cat_/*K*_m_)_cAMP_/(*k*_cat_/*K*_m_)_cGMP_ factor of ∼2. cAMP was found as the best substrate tested, mainly due to the about 2-fold greater *k*_cat_ value as compared to bis-*p*NPP and cGMP. In all cases, the protein had an absolute requirement for Mn^2+^ (maximum activity was achieved when [MnCl_2_] > 5 mM). The apparent dissociation (K_d_) constant for Mn^2+^, calculated from the dependence of phosphodiesterase activity of HP1042 on Mn^2+^ concentration, was determined to be K_d_ of 2.26±0.15 mM. With bis-*p*NPP and cAMP as substrates, HP1042 demonstrated maximum activity at pH from 4.0 to 4.5, and at temperatures ranging from 30 to 40°C (Figure 1). According to activity features, the protein HP1042 should be considered as a 3',5'-cyclic-nucleotide phosphodiesterase (EC 3.1.4.17).

*HP1079* - HP1079 is annotated as a “hypothetical protein” with a predicted mass of 42.9 kDa, possessing an ATPase domain and observed to interact with urease [25]. In this assay, it was found to be able to bind to nano-particles coated with both ATP and GTP with most efficient capture by GTP-coated nano-particles. However, *k*_cat_/*K*_m_ was found to greater for ATP than GTP by a factor of ∼7.5 (Table 1). Hydrolysis of other nucleotides, CTP, GTP, TTP and ADP, were also studied, but the activity levels were too low to be determined adequately. We further analyzed different divalent metal ions (Mg^2+^, Cu^2+^, Co^2+^, Zn^2+^, Ca^2+^, Mn^2+^ and Ni^2+^ - 8 mM each) in an ATPase assay to define the cofactor requirement of HP1079. Only Ca^2+^ was found to affect enzyme activity, but contradictory results were observed with the native protein (slight increase in ATPase activity) and the recombinant protein (inhibition by Ca^2+^), which require further clarification. With both substrates, HP1079 demonstrated maximum activity at pH from 5.5 to 6.5 (being optimal at pH 6.0), and 45°C (Figure 1). Therefore, the protein HP1079 should be considered as an ATPase/GTPase.

*HP0267* - HP0267 is annotated as a “chlorohydrolase” with a predicted mass of 45.5 kDa, and a protein domain Amidohydro_1 from the amidohydrolase family. HP0627 protein was isolated using nano-particles coated with adenine. The native protein was tested for its ability to hydrolyze adenine and a chloro- derivative, 6-chloroadenine, as well as adenosine (Table 1). The protein did show a high affinity (*K*_m_ of 0.32±0.19 mM) and activity (*k_cat_* of 1101±151 min^−1^) for adenosine. A very weak activity with adenine was detected, and a reduction in the catalytic efficiency with this substrate was mainly due to a 2330-fold reduction in the *k*_cat_ as compared to adenosine. No activity toward chloroadenine was observed. Optimal activity with adenosine was observed around 45°C and pH 7.5, making it the only protein of the group tested to show greatest activity at neutral or alkaline conditions (retaining > 70% maximum activity at pH 9.0, but with only 6% activity at pH 4.5). However, for the purified recombinant protein, biochemical activity was only observed using adenosine as a substrate, but not for adenine. This was observed using two different assay methods, both of which produced reasonable *K*_m_ and *K*_cat_ values when compared to other characterized enzymes (Table 1) [26]. However, the ratio *K*_cat_/*K*_m_ is lower for the recombinant protein than previous observations. While the precise reasons for the differences in activity are unclear, there does appear to be slight differences in the catalytic ability of the native and recombinant proteins, even after confirmation of the cloned sequence, and they are currently being investigated. We tentatively suggest that HP0267 be considered as an “*adenosine deaminase*” (EC 3.5.4.4), with slight ability to hydrolyze adenine (EC 3.5.4.2 - and see Discussion).

*HP0310 -* The protein product associated with HP0310 has previously been identified as a peptidoglycan deacetylase PgdA [27], and shown to contribute to bacterial survival [28], [29]. A crystal structure of the protein [30] reveals a structure compatible with this activity, though notes a smaller than expected substrate binding groove as crystallized. In our assay, this protein was isolated using nano-particles coated with acetylated xylan. It was first tested for its ability to hydrolyze acetylated xylan. No activity was observed for the release of reduced sugars when monitored using 3,5-dinitrosalicylic acid (DNS) according to Miller [31], nor by measuring the release of xylose by the D-xylose Rapid Assay kits (Megazyme, Bray, Ireland). By contrast, the enzyme was able to efficiently release acetate groups from acetylated xylan, acetylated chitin and acetylated chitosan (Table 1). Based on the *k*_cat_/*K*_m_ values, the protein functioned more efficiently with xylan than chitin-like products by an order of magnitude. This result can be explained by up to 3.2-fold greater *k*_cat_ values and about 2-fold lower *K*_m_ value for acetylated xylan. Synthetic substrate *p*NPacetate was also efficiently hydrolyzed, at slightly higher efficiency, mainly due to a higher substrate affinity (about 2-fold lower *K*_m_ value) as compared to other acetylated substrates. Using acetylated xylan and *p*NPacetate as substrates, the protein showed maximum activity at 50°C, although it retains about 80% of the activity at 30–55°C, and was active at acidic pH ranging from 5.0 to 6.0, being optimal at pH 6.0 (Figure 1). Taken together with previous work, the protein HP0310 should be considered as a peptidoglycan deacetylase (EC 3.5.1.104), while possessing general “acetylesterase” (EC 3.1.1.6), and specific “acetylxylan esterase” activity (EC 3.1.1.72).

## Discussion

The task of experimentally determining the function of genes for which very little is known is a formidable task, but at the same time, one of significant scientific need. The magnitude of the problem increases daily as ever more DNA sequence is deposited in public databases, and current progress appears slow. For *Mycobacterium tuberculosis* H37Rv, when its genome was sequenced in 1998, there were 912 genes designated as “conserved hypothetical” genes [32]. Today, there are still 906 genes with this annotation. Many of these genes may have well-supported hypotheses or preliminary data regarding their function in the hands of experts engaged in their study, but this information is not readily available to most scientists who might wish to understand the biology of a particular organism. This highlights the need for improved technologies and approaches to experimentally validate protein molecular function.

COMBREX has focused on testing protein function *in vitro*. Within the framework of the Gene Ontology (GO) hierarchy of functional descriptors [33], these functions would be classified under the category of “molecular function.” Predictions of biochemical function are frequently straightforward to test, and results can usually be obtained that support or refute the prediction unambiguously. Furthermore, once a biochemical function is ascertained, it can directly lead to very specific hypotheses of gene function *in-vivo*, allowing an experimentalist to query those functions classified by the GO hierarchy as falling under the heading “biological process.” The full computational methodology underlying the COMBREX framework is described in a subsequent publication (in review).

Our main contribution reported in this paper is deciphering the function of six proteins that previously lacked functional annotation in the clinically important microorganism, *H. pylori* ATCC 26695. Initial hypotheses were generated by a high throughput screen using an affinity-based enrichment methodology to isolate proteins that might bind/act upon the attached substrates. In our case, gold-nano-particles are coated with select substrates, and proteins with affinity toward those substrates can be isolated. Proteins that were isolated in this manner were identified by mass spectrometry, and also used to test directly the biochemical activity of interest.

When assigning biochemical activity to a protein isolated from crude lysates, there is always the risk that the observed biochemical activity is the result of a minor constituent in the isolated sample. For this reason, we cloned each of the identified target genes, expressed them in *E. coli*, purified the overexpressed protein using an epitope tag, and confirmed their biochemical activity *in vitro*. For testing biochemical activity on recombinant proteins, a common pitfall is that cofactors or other protein subunits required for activity will not be present, leading to false negatives. In this paper, we attempted to minimize the combined risks by testing biochemical activity on protein isolated from crude total cell lysates, and on purified recombinant protein. In all cases reported here, we observed essential agreement on the biochemistry of each gene from the two protein sources, with one primary exception. When HP0267 is isolated directly from *H. pylori* lysate, it appears as a single clean band on a polyacrylamide gel (Figure S1), and is observed to hydrolyze adenosine very efficiently and to much lower extent adenine. However with the cloned protein, we were only able to observe activity towards adenosine, but that was with a rather low specificity constant. A recent study presents a metabolic model for purine metabolism in *H. pylori* [34] which accounts for, and observes, both activities, but does not identify the protein(s) responsible. According to the data shown by the isolated protein, we tentatively favor its assignment as an adenosine deaminase, capable of activity towards adenine, allowing for the possibility that both activities may be relevant *in vivo*, and note that genetics will likely soon provide clarification.

We summarize in Table 2 information related to the function of the six genes and the corresponding gene products that were studied in this paper. Prior to these studies, four of the six genes were annotated as “hypothetical protein,” with one protein labeled as “chlorohydrolase” based upon very distant sequence similarity (21% sequence identity) to an experimentally validated gene, atrazine chlorohydrolase from *Pseudomonas* sp. strain ADP. The sixth protein, HP0310, had been previously characterized as a peptidoglycan deacetylase [27]–[30]. While the individual proteins were not named informatively, four of the proteins belonged to protein clusters with descriptive names (HP0267 - PRK08418, HP0310 - CLSK865125, HP1037 - CLSK872355, and HP1042 - CLSK872354), three of which were suggestive to the biochemical activity was eventually observed. Interestingly, these four proteins also possessed annotated domains (Pfam) [35] that in all four cases could have been utilized to predict biochemical activity.

**Table 2:** Genes tested in this paper.

*Locus*	*COMBREX gene annotation*	*COMBREX cluster annotation*	*Protein Domains*	*Function based on isolated native protein and recombinant protein*	*Proposed EC Number*
**HP0267**					
	Chlorohydrolase	Chlorohydrolase	Amidohydro_1	Adenosine deaminase	3.5.4.4
**HP0310**					
	Hypothetical protein	Polysaccharide deacetylase	polysaccharide deacetylase	Acetyl esterase	3.1.1.6
				Acetylxylan esterase	3.1.1.72
**HP0959**					
	Hypothetical protein	Hypothetical protein	-	GTP cyclohydrolase	3.5.4.16
**HP1037**					
	Hypothetical protein	X-Pro aminopeptidase	Creatinase_N	Aminopeptidase	3.4.11.-
			Peptidase_M24		
**HP1042**					
	Hypothetical protein	Putative phospho-esterase RecJ-like	DHH phosphatase	Phosphodiesterase	3.1.4.17
**HP1079**					
	Hypothetical protein	Hypothetical protein	-	ATPase/GTPase	3.6.-.-

One of the remarkable adaptations of *H. pylori* is the ability to thrive in the highly acidic milieu of the gastric environment, with typical pH values < 3.5. *H. pylori* maintains an internal pH ∼8.0 [36], [37] by maintaining high levels of activity of the enzyme urease, which produces ammonia and carbon dioxide, enabling it to buffer large amounts of acid. The pH of the periplasmic space is ∼6.2 over a range of external pHs, providing a proton motive force of greater than 100 mV to enable the generation of ATP [37]. We observe four different profiles for enzyme activity with varying pH. We observe that HP0267 shows maximal adenosine deaminase activity at approximately pH 7.5. HP0310 and HP1079 show maximal acetyl esterase and ATPase activity respectively in a narrow pH range ∼ 6.0. HP1037 exhibits aminopeptidase activity over a broad range of pH, with significant measurable activity at pH < 8.0, and maximal activity at pH < 5.0. Lastly, HP0959 and HP1042 show significant GTP cyclohydrolase and phosphodiesterase activity only at the very acidic pHs of ≤ 4.0. This may indicate protein localization in the cellular interior for HP0267, in the periplasmic space for HP0310 and HP1079, with the enzymes HP0959 and HP1042 either secreted or at the cellular surface.

One of the additional benefits of experimentally validating the function of a protein annotated as “hypothetical protein” is that it then can serve as a source of experimental information that impacts other genes. This is one of the fundamental driving concepts behind COMBREX’s efforts towards establishing a Gold Standard Database of protein function (GSDB), identifying all proteins for which the precise amino acid sequence is known and its biochemical function has been published in the scientific literature. These proteins can then serve as the foundation for the annotation of newly sequenced genes. In Table 3, we highlight the potential reach of the experimental validations reported here. In column 4 we identify 725 proteins in COMBREX that have BLAST *E* values lower (better) than 10^−5^ over 80% of the query and target full length sequence. These proteins are members of clusters (NCBI ProtClustDB [38]) that have a total of over 1200 proteins. Lastly, in the NR database, there are close to 11,000 proteins that have BLAST E values lower than 10^−5^, many of which might have their annotations affected by these observations.

**Table 3:** Extended impact of genes identified.

*Locus*	*UniProt*	*Proposed Function*	*Similar Proteins in COMBREX^a^*	*# Proteins in clusters with similar proteins^b^*	*Blast vs NR^c^*
HP0267	O25046	Adenosine deaminase	148	519	646
HP0310	O25080	Acetylesterase	97	108	1374
HP0959	O25613	GTP cyclohydrolase	121	160	1536
HP1037	O25681	Aminopeptidase	290	353	6867
HP1042	O25683	Phosphodiesterase	29	33	172
HP1079	O25711	ATPase/GTPase	40	56	254
***Totals:***			**725**	**1229**	**10849**

a The number of proteins in COMBREX which have BLAST E values < 1×10^−5^, and alignment over 80% of both the query and target sequence.

b The number of proteins contained in the protein families (from NCBI ProtClustDB [38]) that have at least one BLAST hit, as defined above.

c The number of proteins in the NCBI Non-Redundant database (NR) which have BLAST E values <1×10^−5^.

We also explored the potential consequences of our biochemical assignments on whole-organism *H. pylori* metabolic models - the manually curated ilT341 flux balance model [39], and the biochemical assignments made from automated sequence analysis from Kyoto Encyclopedia of Genes and Genomes (KEGG) [40]. Three activities are not currently represented, even if their activities are expected: adenosine deaminase, EC 3.5.4.4; acetylxylan esterase, EC 3.1.1.72; phosphodiesterase, EC 3.1.4.17. Two of the activities have insufficient specificity, but peptidase and ATPase activity are well represented. Lastly, GTP cyclohydrolase I activity, EC 3.5.4.16, is present, assigned to HP0928, and we have now also assigned that activity to HP0959. Neither protein shares significant sequence similarity, nor do they have common conserved domains, so it is likely that their *in vivo* roles are quite different. The peptidoglycan deacetylase activity of HP0310 has a documented role in conferring resistance to host immune responses via reduced degradation by lysozyme [28]. The acetylxylan esterase activity of this enzyme has not been documented to date for *H. pylori*, but it has been reported in eukaryotic and prokaryotic organisms. Acetylxylan esterases are important enzymes influencing the digestibility of plant cell-wall material by cleaving the ester bonds and thus removing the acetyl moieties from complex carbohydrates. It is possible that this enzyme might play a role during infection, considering that xylan-containing carbohydrate structures are among those commonly consumed by humans [41]. HP1042 was identified as a phosphodiesterase with EC number 3.1.4.17, an activity found widely in the annotations of multi-cellular, eukaryotic, and prokaryotic organisms, and plays an important role in purine metabolism. Purine nucleotide salvage pathways are an integral part of genome scale metabolism, required for utilizing purine compounds from the environment. This pathway has been demonstrated in *H. pylori* [34], but the proteins responsible for adenosine deaminase and adenine deaminase activity were not known. We find that HP0267 has strong adenosine deaminase activity, and much weaker adenine deaminase activity, and could potentially be responsible for one or both of these activities *in vivo*. A protein that possesses adenine deaminase activity exclusively could be a potential drug target, since interference with the purine nucleotide salvage pathway reduces growth, and the activity is not observed in humans.

Ultimately, biological understanding of a protein’s function incorporates information related to the specific role of a protein (its molecular function) and the biological process, or pathway, which its molecular function impacts. That is, most biologists desire information related to both its *in vivo* and *in vitro* role. We point out that once a biochemical or *in vitro* function of a protein is determined, genetic methods, such as phenotype observation on knockout strains, can quickly lead to very specific hypotheses about *in vivo* roles proteins may serve. These types of experiments are frequently amenable to existing high-throughput approaches, placing the onus on the scientific community of developing higher-throughput biochemical approaches in hopes of enabling our knowledge of gene function to keep pace with our ability to identify new genes through DNA sequencing.

## Supporting Information

Figure S1 **SDS-PAGE analysis of Coomassie-stained proteins isolated using derivatized gold nano-particles.** In four of the five instances only a single intense band is observed. In lane 5, two bands were observed, which were identified using mass spectrometry as HP0803 and HP0959. GTP cyclohydrolase activity was observed for HP0959, however HP0803 resisted multiple attempts at cloning and expression in *E. coli*, it was not pursued further. HP0803 only has one know protein domain, DUF3943 (PF13084), and so its reason for binding to the derivatized nano particle coated with guanosine-5'-triphosphate (GTP) and dihydroneopterin triphosphate is unexplained. (Data for HP0310 not shown.).
(DOC)
